# ICP and CPP management before and after 2007: impact on the association between dose of ICP and outcome

**DOI:** 10.1186/2197-425X-3-S1-A441

**Published:** 2015-10-01

**Authors:** S Boeckx, F Guïza, B Depreitere, G Citerio, I Piper, P Jorens, A Maas, MU Schumann, G Van den Berghe, G Meyfroidt

**Affiliations:** 1 Universitary Hospital Leuven, Intensive Care, Leuven, Belgium; 2 Universitary Hospital Leuven, Neurosurgery, Leuven, Belgium; 3 San Gerardo Hospital, Neuroanesthesia and NeuroIntensive Care, Monza, Italy; 4 University of Glasgow, Clinical Physics, Glasgow, United Kingdom; 5 Universitary Hospital Antwerpen, Intensive Care, Antwerp, Belgium; 6 Universitary Hospital Antwerpen, Neurosurgery, Antwerp, Belgium; 7 Kantonsspital Baselland, Anaesthesia and Intensive Care, Bruderholz, Switzerland

## Introduction

In a recent paper, the intracranial pressure-time burden associated with worse outcome in traumatic brain injury (TBI) patients was visualised in a color-coded plot [1]. This color-coded plot illustrates the intuitive concept that episodes of higher intracranial pressure (ICP) can only be tolerated for shorter durations: the transition curve that delineates the duration and intensity of those ICP episodes associated with worse outcome is an approximately exponential decay curve. The study was done in a large prospective multicenter European cohort of patients, including patients from before and after 2007. In 2007, the guidelines on cerebral perfusion pressure (CPP) management in severe TBI have changed [2].

## Objectives

To assess whether CPP management before and after 2007 was different in this European multicenter cohort.

To assess whether the ability to sustain to sustain insults of elevated ICP was different in the most recent cohort.

## Methods

Patients before and after 2007 were assigned to different cohorts, and standard statistical tests were used to compare the baseline patient characteristics. The mean CPP before and after 2007 was determined. The transition curve, as described in [1], was redrawn in the after 2007 subgroup and compared to that reported in [1].

## Results

**Figure 1:**
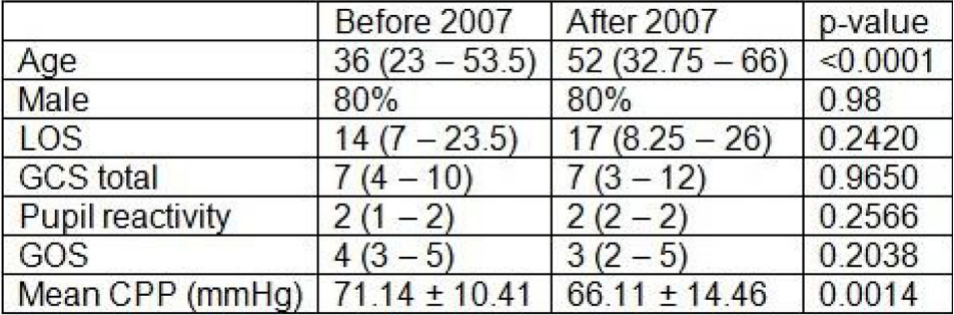
**Comparison of baseline characteristics.**

**Figure 2:**
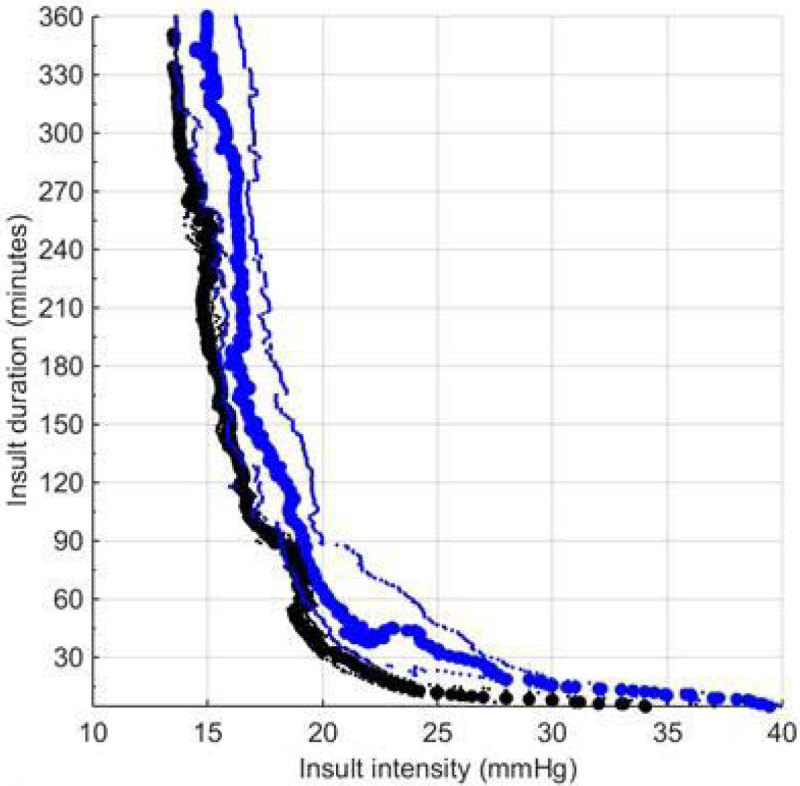
[Comparison of ICP insult transition curve] Comparison of ICP insult transition curve for ‘after 2007’ cohort (blue) and ICP insult transition curve of [1] (black). Thick lines are 0-correlation curves and thin lines -0.2 and 0.2 correlation curves respectively.

Of the 261 patients, 166 patients (admitted between 2003 and 2005) were assigned to the 'before 2007' cohort, and 95 patients (admitted between 2010 and 2013) to the 'after 2007' cohort. Baseline characteristics between both cohorts are compared in Figure 1. Patients in the after 2007 cohort were significantly older (p-value < 0.0001). The mean CPP was significantly lower after 2007 (66.11 mmHg ± 14.46 mmHg vs. 71.14 mmHg ± 10.41 mmHg, p-value= 0.0014). In the after 2007 cohort, there was a shift to the right of the transition curve (Figure 2). However, the 'after 2007' cohort was too small to conclude whether this represents a better tolerance for increased ICP insults, as the region of low correlation (used to define the transition curve) is much broader.

## Conclusions

In this multicenter European cohort, lower CPP levels were applied after 2007, in accordance with the BTF guidelines. When plotting the ICP time-pressure burden plot after 2007, the approximately exponential transition curve remained, albeit with shifts in the thresholds. There is not enough statistical power to assess whether the change in TBI management after 2007, has been able to influence the time and pressure thresholds at which secondary injury occurs.

## Grant Acknowledgements

Foundation for Scientific Research Flanders (FWO) (G. 0904.11). Senior clinical investigator, FWO to Geert Meyfroidt (1846113N). Methusalem program, Flemish Government to Greet Van den Berghe (METH/08/07).
